# Comparison of Economic Evaluation Methods Across Low‐income, Middle‐income and High‐income Countries: What are the Differences and Why?

**DOI:** 10.1002/hec.3312

**Published:** 2016-01-17

**Authors:** Ulla Kou Griffiths, Rosa Legood, Catherine Pitt

**Affiliations:** ^1^ Department of Global Health and DevelopmentLondon School of Hygiene and Tropical MedicineLondonUK; ^2^ Department of Health Services Research and PolicyLondon School of Hygiene and Tropical MedicineLondonUK

**Keywords:** cost‐effectiveness, cost‐benefit, review, low‐income and middle‐income countries, research methods

## Abstract

There are marked differences in methods used for undertaking economic evaluations across low‐income, middle‐income, and high‐income countries. We outline the most apparent dissimilarities and reflect on their underlying reasons. We randomly sampled 50 studies from each of three country income groups from a comprehensive database of 2844 economic evaluations published between January 2012 and May 2014. Data were extracted on ten methodological areas: (i) availability of guidelines; (ii) research questions; (iii) perspective; (iv) cost data collection methods; (v) cost data analysis; (vi) outcome measures; (vii) modelling techniques; (viii) cost‐effectiveness thresholds; (ix) uncertainty analysis; and (x) applicability. Comparisons were made across income groups and odds ratios calculated. Contextual heterogeneity rightly drives some of the differences identified. Other differences appear less warranted and may be attributed to variation in government health sector capacity, in health economics research capacity and in expectations of funders, journals and peer reviewers. By highlighting these differences, we seek to start a debate about the underlying reasons why they have occurred and to what extent the differences are conducive for methodological advancements. We suggest a number of specific areas in which researchers working in countries of differing environments could learn from one another. © 2016 The Authors. *Health Economics* published by John Wiley & Sons Ltd.

## 1 Introduction

Economic evaluation (EE) is defined as a ‘comparative analysis of alternative courses of action in terms of both their costs and their consequences’ (Drummond *et al.*, 2005). EEs aim to identify how to employ scarce resources most efficiently to improve health, thereby achieving ‘value for money’. The number of EE studies has increased substantially over the past three decades, both in high‐income (Neumann *et al.*, 2009, Greenberg *et al.*, 2010) and in low‐income and middle‐income settings (Suhrcke *et al.*, 2012). In a bibliometric analysis published in this special issue, Pitt *et al*. (2016) found that more than 100 EEs were published every month during 2012–2014.

The quality of EEs has been assessed in several reviews, which have generally concluded that a relatively large proportion of studies do not adhere to standard guidance (Jefferson *et al.*, 2002, Gerard *et al.*, 1999, Neumann *et al.*, 2000). Consequently, several checklists that stipulate requirements of what a high‐quality study should contain and report have been developed (Husereau *et al.*, 2013, Drummond and Jefferson, 1996). Reviews of EEs often use these checklists for quality assessments (Fang *et al.*, 2011, Griffiths and Miners, 2009). However, to date no review has directly compared the methods used for EE across country income groups.

In this paper, we identify some of the most important methodological differences between EEs in low‐income, middle‐income, and high‐income countries, reflect on the reasons for the dissimilarities and discuss implications for research standards.

## 2 Methods

### 2.1 Identification of areas of methodological difference

We identified ten methodological areas in which we expected to find marked differences across settings. These were partly based on the Consolidated Health Economic Evaluation Reporting Standards checklist (Husereau *et al.*, 2013) and from our experience working in low‐income, middle‐income and high‐income countries. The ten areas are as follows: (i) availability of guidelines; (ii) research questions; (iii) perspective; (iv) cost data collection methods; (v) cost data analysis; (vi) outcome measures; (vii) modelling techniques; (viii) cost‐effectiveness thresholds; (ix) uncertainty analysis; and (x) applicability. In Section 3, we describe each methodological topic in turn. Our perceptions of the underlying reasons for the differences are given in the discussion.

### 2.2 Cross‐sectional analysis of methodological differences across country income groups

To quantify the degree of methodological variation between country income groups, we conducted a cross‐sectional analysis of a representative sample of recently published EEs. We randomly selected a stratified sample of 150 studies from a comprehensive database of 2844 EEs published between 1 January 2012 and 3 May 2014 (Pitt *et al.*, 2016). Pitt and colleagues constructed this database by systematically searching 14 literature databases, screening more than 15 000 unique records and retaining only primary research articles, which either produced a summary measure of efficiency (such as an incremental cost‐effectiveness ratio, incremental net benefit, probability of cost‐effectiveness given a threshold or a cost‐effectiveness plane or acceptability curve) or demonstrated strict dominance. Articles described as cost‐effectiveness or cost‐benefit analyses, but not meeting these inclusion criteria, were excluded.

Fifty articles were sampled from each of three country income groups: low‐income and lower‐middle‐income countries (LLMICs, gross national income (GNI) per capita < US$ 4083), upper‐middle‐income countries (UMICs, US$ 4086 < *x* < US$ 12 615) and high‐income countries (HICs, GNI per capita > US$ 12 616) (World Bank, 2014). We chose to combine low‐income and lower‐middle income countries into one group because the overall number of studies in each group were small and we believe that the capacity for EE research in these countries is comparably low. Multi‐country studies spanning more than one of the three income groups were excluded, while multi‐country studies that examined countries within only one of the three income groups were included. Sampling was performed using Excel's random number generator to assign a random value between 0 and 1 to all articles, and then selecting the 50 articles within each group with the highest values. We extracted data from the full texts of the 150 articles using a standardised data extraction tool reflecting the ten methodological areas. We then compared findings in these methodological areas across income groups and performed a logistic regression to estimate the odds of income group being a predictor of the chosen method. Full references of the 150 selected studies are included in the web annex.

### 2.3 Other sources of evidence

For each of the ten methodological areas, we also examined other relevant evidence. In particular, we identified country‐specific EE guidelines on the website of the International Society for Pharmacoeconomics and Outcomes Research (International Society for Pharmacoeconomics & Outcomes Research, 2015).

## 3 Results

### 3.1 Published guidelines

In HICs, EEs have frequently been conducted as part of health technology assessments (HTAs). This is ‘a form of policy research that examines short‐term and long‐term consequences of a healthcare technology, including evidence of safety, efficacy, patient‐reported outcomes, cost and cost‐effectiveness’ (International Society for Pharmacoeconomics & Outcomes Research, 2003). HTA originated in the USA in the 1960s, but the approach has especially been embraced in Western Europe (Neumann, 2009). During the 1980s, government HTA offices were established in several European countries, and these gradually began to publish HTA guidelines, which included specific guidance on the conduct and reporting of EEs (O'Donnell *et al.*, 2009, Philips *et al.*, 2004). Importantly, HTA with an EE component has become compulsory in several countries when assessing whether a new pharmaceutical product should be reimbursed by publicly funded healthcare systems. The requirement to demonstrate that new products are cost‐effective as well as of good quality, effective and safe has been described as the ‘fourth hurdle’ (Rawlins, 2012).

As of mid 2015, 50% of HICs, 20% of UMICs and 1% of LLMIC had official EE guidelines (Table 1). While an application of these guidelines was only a recommendation in ten countries, it was a requirement for reimbursement or inclusion in publicly provided services in the remaining 30. Australia was the first HIC to publish guidelines in 1992, joined most recently by Italy, Croatia, Slovak Republic and Finland in 2011. The median year for the first edition of guidelines in HICs was 2003. In 2003, Cuba was the first UMIC to publish HTA guidelines, and between 2008 and 2014, an additional eight UMICs also did this: Mexico (2008), Thailand (2008), Brazil (2009), South Africa (2010), China (2011), Malaysia (2012), Hungary (2013) and Colombia (2014). Egypt is the only LLMIC with official guidelines, issued in 2013.

**Table 1:** Availability of official economic evaluation guidelines

Country income group	Number of countries	Number of countries with methodological recommendations	Number of countries with methodological requirements for reimbursement	Number of countries with any economic evaluation guidelines	Median year of first edition
Low‐income and lower‐middle income	84	0 (0%)	1 (1%)	1 (1%)	2013
Upper‐middle income	51	3 (6%)	6 (12%)	9 (18%)	2011
High‐income	60^a^	7 (12%)	23 (38%)	30 (50%)	2003
Total	195	10 (5%)	30 (15%)	40 (21%)	2006

Source: ISPOR website (International Society for Pharmacoeconomics & Outcomes Research, 2015).

a Scotland and Taiwan are counted as independent countries as they have their own guidelines.

Income groups reflect World Bank classifications (2014).

Instead of national guidelines, researchers working in LLMICs have tended to use guidance published by international organisations, in particular, the World Health Organization (WHO). These guidelines have often been produced for disease‐specific programmes, such as for vector control and immunisation (Phillips *et al.*, 1993, Walker *et al.*, 2010). However, several of them have focused more on estimation of intervention costs than on effect measurement (Kumaranayake *et al.*, 2000, World Health Organization, 1988). In 2003, the WHO published guidelines for ‘Generalised Cost‐effectiveness Analysis’ (Edejer *et al.*, 2003), but this method has not been widely applied outside this organisation. During 2013–2014, the Bill and Melinda Gates Foundation funded development of a ‘reference case’ with the objective of increasing the consistency of EE methodology for studies undertaken in low‐income and middle‐income countries (NICE International, 2014, Santatiwongchai *et al.*, 2015). The reference case is founded on 11 key principles to guide the planning, conduct and reporting of EEs. Some of these principles are quite specific compared with the Consolidated Health Economic Evaluation Reporting Standards checklist, which only lists items that should be included when reporting EEs. For example, the Bill and Melinda Gates Foundation reference case recommends using an outcome measure that is generalisable across disease states and to include a budget impact analysis as well as an equity analysis (NICE International, 2014).

### 3.2 Research questions

In our cross‐sectional analysis, we found that the majority (56%) of LLMIC studies examined infectious diseases, as did a large proportion (39%) of UMIC studies, compared with just 14% of HIC studies (Tables S1–S3 in Supporting Information). Chronic conditions, defined as diseases not passed from person to person and of long duration, were studied in 22% of LLMIC studies, 62% of UMIC studies and 80% of HIC studies. Other health problems, including malnutrition, obstetric emergencies, iodine deficiency and fractures, were studied in 22% of LLMIC studies and in just 2% and 6% of UMIC and HIC studies, respectively. The logistic regression showed that the odds of studying a chronic disease were 3.83 greater for each increase in income group (Table 3). These trends largely mirror the 2011 global burden of disease estimates, although in all income groups, infectious diseases are studied in a substantially higher proportion of studies than their share of the disease burden. Infectious diseases accounted for 33% of the disease burden in LLMICs, 11% in UMICs and 4% in HICs. Chronic conditions, by our definition, constituted 41% of the disease burden in LLMICs, 71% in UMICs and 84% in HICs (World Health Organization, 2014).

The types of interventions evaluated varied considerably by income group. While new drugs were evaluated in 36% and 24% of the UMIC and HIC papers, respectively, none of the 50 studies set in LLMICs evaluated a new drug (Table 2). Instead, 26% of LLMIC papers assessed an increased use of a mature drug already prevailing in the essential drug list of the country, and 42% evaluated the introduction of new services. New services in LLMICs were often relatively complex interventions, such as school support for orphan girls to prevent human immunodeficiency virus infection (Miller *et al.*, 2013), mobile phone text messages to health workers with reminders about malaria treatment procedures (Zurovac *et al.*, 2012) and increased leprosy case detection (Ezenduka *et al.*, 2012).

**Table 2:** Types of interventions evaluated in sample of 150 articles

Type of intervention	LLMIC (%)	UMIC (%)	HIC (%)
New service	42	8	18
Increased use of old drug	26	14	14
New vaccine	14	16	6
Surgery	10	6	12
Nutrition	4	4	0
Screening	2	14	16
Diagnostics	2	2	10
New drug	0	36	24

LLMIC, low‐income and lower‐middle income countries; UMIC, upper‐middle‐income countries; HIC, high‐income countries.

### 3.3 Perspective of analysis

The perspective taken in an EE determines whose costs should be taken into account. There are generally two main perspectives to consider, the health sector (i.e. provider or third party payer) and society. When using a health sector perspective, costs borne by patients and their families or by other sectors are excluded. In the societal perspective, all costs are included irrespective of who pays. Time costs, also referred to as productivity costs, should also be included in the societal perspective.

The government health sector perspective is required in the primary analysis of 55% of the EE guidelines from HICs and UMICs (International Society for Pharmacoeconomics & Outcomes Research, 2015). The US guidelines recommend a payer perspective in primary analysis and societal in secondary analysis, reflecting the country's insurance‐based health care financing (Academy of Managed Care Pharmacy, 2012). In contrast, the societal viewpoint is consistently recommended as the preferred perspective in guidelines used by LLMIC researchers (Edejer, 2003, Walker *et al.*, 2010). However, the Egyptian guidelines only require a government health sector perspective (Elsisi *et al.*, 2013).

The guideline recommendations were to some extent reflected in the findings of our cross‐sectional analysis. A government health sector perspective was taken in 70% of the HIC studies, 66% of the UMICs and 60% of the LLMICs. A societal perspective was taken in 18%, 20% and 36% of the studies from HICs, UMICs and LLMICs, respectively. A payer's perspective was used in 12% of both HIC and UMIC studies and in none of the LLMIC studies. The patient's perspective was used in 2% of the UMIC studies and 3% of the LLMIC studies, but in none of the HIC studies. The differences between income groups in the proportion of studies employing a government health sector perspective were not, however, statistically significant (*p* = 0.295, Table 3).

**Table 3:** Results of logistic regression to test for differences in methodological areas across country income groups

	Methodological area	Binary variable	*n*	LLMIC (%)	UMIC (%)	HIC (%)	Odds ratio	*P*‐value
1	Availability of guidelines	Guidelines available	195	1	18	50	6.66	<0.001
2	Research question	Chronic disease studied	150	80	62	22	3.83	<0.001
3	Perspective	Government perspective used	150	60	66	70	1.25	0.295
4	Cost data collection methods	Ingredients approach	150	68	30	10	0.22	<0.001
5	Cost data analysis	Patient‐level cost data analysed	32	44	75	55	1.18	0.711
6	Outcome measure	QALYs	150	28	42	66	2.24	<0.001
7	Modelling technique	Markov model	150	16	38	50	2.21	<0.001
8	Cost‐effectiveness threshold	WHO (GDP or GNI‐based)	150	52	46	6	0.32	<0.001
9	Uncertainty analysis	Probabilistic sensitivity analysis	150	34	42	72	2.22	<0.001
10	Applicability	At least one local author	150	82	82	98	2.17	0.021

LLMIC, low‐income and lower‐middle income countries; UMIC, upper‐middle‐income countries; HIC, high‐income countries; QALYs, quality adjusted life years; WHO, World Health Organization; GDP, gross domestic product; GNI, gross national income.

### 3.4 Cost data collection methods

Costing methods should follow from the aim of the analysis and the availability of data (Mogyorosy and Smith, 2005). At one end of the spectrum, the ingredient approach, or micro‐costing, entails direct measurement of resources and their associated unit costs. If relevant for the analysis, patient‐specific data collection is preferable because it allows for more detailed analysis of variation in costs and cost‐effectiveness. At the other end of the spectrum, costs can be estimated by using reference costs derived from a non‐patient‐specific source, such as diagnosis‐related groups based on national administrative databases. This is referred to as the gross costing or top‐down method. Gross costing is usually faster and cheaper for the analyst than micro‐costing, but may be less accurate because relatively large resource units are measured. Micro‐costing may be more reliable and precise, but it can be expensive and not always practical (Mogyorosy and Smith, 2005). Moreover, because of challenges in data collection, micro‐costing studies do often not incorporate costs that are incurred jointly with other diseases or interventions across the health facility or wider health system, which can lead to marked underestimation of economic costs (Cunnama *et al.*, 2016).

There were clear differences between country income groups regarding costing methods. The ingredients approach was used as the sole method in 68% of studies set in LLMICs, compared with 30% and 10% of UMIC and HIC studies, respectively. In contrast, reference costs (sometimes mixed with some micro‐costing) were used in 78% of HICs and 45% of UMICs, but not in any LLMIC studies. Secondary data, such as from other peer‐reviewed articles, were used in 28% of LLMICs, 23% of UMIC studies and 12% of HIC studies. Patient invoices from user fees were applied as a proxy for costs in two studies, one set in Kenya (Shade *et al.*, 2013) and one set in Thailand (Muangman and Totanarungroj, 2012).

### 3.5 Cost data analysis

When patient‐specific cost data are collected, as is usual when EE studies are undertaken alongside a randomised clinical trial, researchers have the opportunity to conduct statistical analysis on patient‐specific cost data. This might include an analysis of uncertainty, adjusting for confounders and subgroup analysis. EE guidelines in general do not, however, give detailed guidance on how patient level data should be analysed, although the guidance of United Kingdom's (UK) National Institute for Health and Care Excellence (NICE) makes clear that patient‐level data is preferable to facilitate subgroup analysis (National Institute for Health and Care Excellence, 2013).

The proportion of studies conducted alongside a clinical trial was highest in LLMICs, with 32% of studies. Only 14% of studies from both UMICs and HICs were conducted alongside a clinical trial. The proportion of these studies presenting statistical analysis of patient cost data was 44% in the LLMIC group, 75% in UMICs and 55% in HICs. Hence, about half of papers across all income groups with patient cost available from clinical trials did not present a detailed analysis of these data. Because of the small sample size, the odds ratio of 1.18 was not statistically significant (Table 3).

### 3.6 Outcome measures

The choice of outcome measure is important as it largely dictates the usefulness of the analysis. There is broad agreement between health economists that it is preferable to use a composite outcome measure, which combines length and quality of life into a single metric (Ramsey *et al.*, 2005, NICE International, 2014). Disability adjusted life years (DALYs) and quality adjusted life years (QALYs) are the most commonly used.

Composite measures facilitate comparisons among interventions against all health conditions, whereas outcomes measured in natural units, such as ‘number of diabetes cases averted’, only permit comparisons with findings from studies using the same, limited metric. However, measures in natural units can be useful for budget allocations within a disease‐specific programme. In clinical trials, effects are often measured by intermediate or surrogate outcomes, such as ‘change in glycated haemoglobin’, to reduce the trial's duration and expense. Predicting long‐term health effects in terms of length and quality of life from surrogate measures thus presents a key challenge requiring decision analytic modelling (Ramsey *et al.*, 2005).

Calculating QALYs or DALYs entails four main steps: (i) describing the health state; (ii) developing preference scores or weights for the health state; (iii) determining the time spent in each health state; and (iv) combining the information. Development of preference scores or weights for the health state is the most methodologically challenging aspect and an area where DALYs and QALYs particularly differ (Gold *et al.*, 2002). Preference scores for QALYs are derived by asking patients to complete questionnaires about their overall health status. These data are scored using a multi‐attribute scoring function based on direct preference measurement from random samples of the general population of the respective country. Two types of preference‐scored, multi‐attribute health status classification systems are in widespread use: the EuroQol EQ‐5D and the Health Utility Index (Torrance *et al.*, 2002). Multi‐attribute scoring functions for the EQ‐5D system are available for 20 HICs, four UMICs (Argentina, China, Hungary and Thailand) and two LLMICs (Armenia and Zimbabwe) (EuroQol, 2015).

Disability adjusted life years were originally developed to facilitate comparisons of disease burden between countries. The same disability weights are therefore used for all settings. In the original 1996 WHO Global Burden of Disease study, 237 different disability weights were published (Murray, 1996). These were derived by using the person trade‐off approach with a group of experts (Fox‐Rushby, 2002). The most recently updated disability weights have been obtained from population surveys in five countries (Salomon *et al.*, 2012).

In our cross‐sectional analysis, QALYs were used in 66% of studies in HICs, 42% of UMICs and 28% of LLMICs. DALYs were used in 2%, 18% and 30% of the HIC, UMIC and LLMIC papers, respectively. For each level of increase in income group, the odds of using QALYs in the EE were 2.24 greater, and this relationship was statistically significant (Table 3). Combining these figures, we find that 68% of HIC, 60% of UMIC and 58% of LLMIC studies used a composite outcome metric (i.e. DALYs or QALYs), indicating a very small difference across income groups, which was not statistically significant (odds ratio = 1.24, *p* = 0.304). The outcome measures in natural units included ‘diarrhoea case averted’ (Puett *et al.*, 2013) and ‘detection of individual with high cardiovascular risk’ (Selvarajah *et al.*, 2013).

### 3.7 Modelling techniques

Modelling is useful for extrapolating the time horizon to a lifetime and synthesising all available data (Sculpher *et al.*, 2006). A number of modelling techniques exist; the most appropriate depends on the research question. For simple questions, a decision tree may be sufficient. Markov models are especially helpful where there are repeat events over time, such as in chronic conditions. For infectious diseases, transmission dynamic models can account for herd immunity and other indirect effects (Drake *et al.*, 2015). While EE guidelines are generally not specific about which type of modelling approach should be used, the need for modelling is clear given recommendations of a lifetime horizon, which is normally unavailable from observed data.

In our sample, there were marked differences among settings, both in the use of modelling *per se* and the types of model used. Nearly half of LLMIC studies (48%) had no model or the type was not clear from the description in the paper, whereas this was the case in 34% and 14% of papers from UMICs and HICs, respectively. The most common approach in HICs was the Markov model (56% of the 43 models), which may reflect that the majority of HIC papers assessed chronic conditions. None of the HIC studies used transmission dynamic models, whereas 10% and 6% used such models in UMICs and LLMICs, respectively. Microsimulation approaches were used in 12% of HIC, 6% of UMIC and 4% of LLMIC articles.

### 3.8 Cost‐effectiveness thresholds

When the comparison of interventions in an EE does not reveal dominance (i.e. that one intervention is both more effective and less costly than the other), decision makers must determine whether the additional costs incurred by one intervention relative to another are worth its additional benefits. In theory, all potential interventions could be ranked from highest to lowest priority, taking into account cost‐effectiveness and other criteria, and designated for implementation in order of priority until the overall budget constraint is reached. In practice, a variety of cost‐effectiveness threshold values have been used to determine whether an intervention is likely to be cost‐effective, without directly comparing with every possible alternative use of the resources.

In our representative cross‐section, 80% of HIC, 60% of LLMIC and 52% of UMIC studies referred to a threshold. Of these, 87% and 88% of articles set in LLMICs and UMICs, respectively, referred to the WHO thresholds, compared with only 8% in HICs. The WHO threshold defines ‘highly cost‐effective’ interventions as those costing less than per capita gross domestic product (GDP) and ‘cost‐effective’ as those costing less than three times per capita GDP (World Health Organization, 2015). The remaining four LLMIC studies used arbitrary thresholds or compared against interventions that were already implemented. Two of the three UMIC studies that used another threshold were set in Thailand and used Thailand's national threshold of GNI per QALY gained (Kulpeng *et al.*, 2013, Rattanavipapong *et al.*, 2013).

Of HIC studies employing a threshold, 28% (*n* = 11) used the NICE thresholds of £20 000 or £30 000 per QALY gained (National Institute for Health and Care Excellence, 2013). Of these, seven were from the UK, two were multi‐country studies from the UK and another European country, one was entirely set in Italy and another in the USA. In HICs, 65% (*n* = 26) used other thresholds. Seven of the 14 papers from the USA used US$ 50 000 per QALY gained, and two identified thresholds based on specific health outcomes: £100 000 per timely diagnosis of congenital heart disease (Ewer *et al.*, 2012) and US$ 50 000 per alloimmunisation event averted (Kacker *et al.*, 2014).

As many as 96% of LLMIC, 82% of UMIC and 78% of HIC studies concluded that a new or additional intervention would be highly cost‐effective or cost‐effective. It can be argued that thresholds set so high that nearly all possible interventions are considered ‘cost‐effective’ cannot contribute effectively to priority setting. Shillcut and colleagues demonstrated that the seemingly arbitrary thresholds used in some HICs (including the NICE threshold of £30 000 per QALY gained) were in fact consistent with approximately twice per capita GNI. They also argued that GNI and GDP‐based thresholds ‘may reinforce wide global inequities in health and wealth’, ‘lead to total budgetary costs that are currently not sustainable’ and that ‘valuing health according to income ignores the other dimensions of life that can be argued to have utility’ (Shillcutt *et al.*, 2009). In line with this critique, Drake has argued for a minimum ‘transnational’ threshold, on the basis that ‘using country‐specific thresholds in cross‐country analysis conflate cost‐effectiveness with affordability’ (Drake, 2014).

The widespread use of the NICE thresholds in UK analyses is clearly driven by NICE's explicit requirement, which also makes clear that the NICE threshold is intended to reflect the fixed budget constraint of the National Health Service (NHS). However, recent empirical work using routine cost and health outcomes data from the NHS estimated a threshold of £12 936 per QALY and indicated that even this far lower estimate was likely to overestimate affordability (Claxton *et al.*, 2015). The use of threshold values that are likely to be unaffordable is thus common to all countries.

### 3.9 Uncertainty analysis

The impact on the result of parameter and structural uncertainties should be assessed in sensitivity analysis. The simplest option is to vary one or more parameter estimates at a time and re‐run the evaluation in a deterministic sensitivity analysis. However, this does not allow for an overall assessment of the degree of uncertainty or most probable estimates. Probabilistic sensitivity analysis (PSA) is undertaken by specifying parameters as distributions rather than point estimates and simultaneously varying all parameter estimates in a Monte Carlo simulation. Thus, PSA does not only provide estimates of mean expected costs and effects but also accompanying uncertainty ranges, typically expressed as cost‐effectiveness acceptability curves or frontiers (Briggs, 2001).

In our cross‐sectional analysis, there were differences across settings as to whether sensitivity analysis was conducted at all and the type of analysis performed. Across LLMICs, UMICs and HICs, 22%, 16% and 4% of papers, respectively, did not perform any sensitivity analysis. PSA was significantly more common in HICs where it was undertaken in 72% of all 50 studies, compared with 42% in UMICs and 34% in LLMICs (Table 3).

As all guidelines recommend exploration of uncertainty (Drummond *et al.*, 2005), the complete lack of sensitivity analysis in one‐fifth of LLMIC studies constitutes a weakness. The NICE methods guidelines recommend PSA (National Institute for Health and Care Excellence, 2013), which may in part explain its high usage in HICs; however, not all guidance contains this recommendation.

### 3.10 Applicability

Applicability refers to the extent to which results of EEs are relevant to local contexts and used by policymakers in decision‐making. In countries where EE is a requirement for pharmaceutical reimbursements, they are clearly being used for decision‐making. However, this criterion only captures decisions at the highest level, and it does not show the extent to which conducted studies are aligned with public health priorities.

The impact of EEs on healthcare decision makers is believed to be limited (Thurston *et al.*, 2008). Hoffmann and Graf von der Schulenburg surveyed decision makers from nine European countries and found reasons for results of EEs not being widely used included institutional dimensions, such as difficulties in transferring budgets, and lack of credibility of studies funded by pharmaceutical companies. A UK study found that while decision makers believe that EEs are valuable in principle, their usefulness is limited because they do not always apply to their settings (Hoffmann *et al.*, 2002). Moreover, UK NHS decision makers have been found to either not understand health economics outcome statements, such as incremental cost‐effectiveness ratios (ICERs) and QALYs, or consider these to be irrelevant (Duthie *et al.*, 1999).

A systematic review on perceptions of health policymakers on their use of evidence found that the most commonly mentioned facilitator of using research evidence in policymaking was ‘personal contact between researchers and policymakers’, and similarly, the most commonly mentioned barrier was ‘absence of personal contact between researchers and policymakers’ (Innvaer *et al.*, 2002). A study on decision‐making for vaccines similarly found locally generated evidence to be critical (Burchett *et al.*, 2012). Hence, policymakers are more inclined to use study results if they or an institution they know is in direct contact with the researchers. As an indicator of potential applicability, we explored whether studies were locally funded and whether authors were based in the study country. In our cross‐sectional sample, 74% of HIC studies were locally funded, as were 60% of UMIC studies, but only 20% of LLMIC studies. We found that 98% of HIC studies included an author affiliated with an institution located in at least one of the countries studied, compared with 82% in both UMICs and LLMICs. This difference was statistically significant (*p* = 0.021) (Table 3). As authorship is important both for capacity development and for fostering relevant and applicable EEs, it is encouraging that a relatively high proportion of LLMIC studies included at least one local author, despite the low proportion of domestic funding. This finding does not, however, take into account author order or the nature of contributions from different authors. Furthermore, the local authorship criteria is likely to be less relevant for LLMIC studies funded and used by international donors, such as the Global Fund and the Bill and Melinda Gates Foundation (Santatiwongchai *et al.*, 2015).

## 4 Discussion

We found that methods used for EEs particularly vary between income groups with regard to cost data collection, outcome measures, modelling techniques, cost‐effectiveness thresholds and uncertainty analysis. There were also significant differences regarding availability of guidelines and research questions, but these two aspects reflect contextual differences and not chosen research methods. While EEs also share many common features across income groups, we have chosen to focus on differences, which have previously been noted anecdotally but neither quantified nor addressed. By highlighting these differences, we aim to start a debate about the underlying reasons for why they have occurred and to what extent the differences are conducive for methodological advancements. Here, we discuss our perceptions of some of the underlying reasons for methodological differences.

Firstly, genuine *differences between settings, diseases, and health care systems* rightly affect methodological choices. While the specific health areas studied do not and would not be expected to correlate perfectly with burden of disease, the greater focus on infectious diseases in EEs from LLMICs reflects real differences in epidemiological profiles, which influence the appropriate type of modelling. Differences in the nature of interventions evaluated may also to some extent appropriately reflect differing health system contexts; the tendency of EEs in HICs to focus on relatively expensive new technologies and of LMICs to focus on complex health systems interventions may reflect the greater supply and demand constraints on LLMIC health systems (Vassall *et al.*, 2016). Moreover, the large majority of new drugs are produced with HICs as the target market, and it is important to assess their value‐for‐money (Trouiller *et al.*, 2002).

The appropriate perspective may also be context specific. The difference between government and societal perspectives will invariably be smaller when the bulk of health care costs are covered by the government in settings with universal health care coverage compared with countries with limited social security. Nonetheless, patients inevitably incur at least some costs during illness; productivity costs are especially important for chronic conditions. Only 36%, 20% and 18% of studies from LLMICs, UMICs and HICs in our cross‐sectional sample took a societal viewpoint, respectively. Failure to capture ‘true’ efficiency by including costs from all payers is thus a shortcoming of studies from all income groups, but the consequences are starkest in settings and for diseases where patients incur a relatively large proportion of costs. A key reason for choosing a government perspective is most likely a desire for less data collection; a societal perspective would often require patient interviews, which invariably increase study expenses.

Secondly, there are different *capacities within the health sector*. For example, the lack of cost accounting systems in LLMICs often necessitates an ingredients approach to costing or a combination of primary cost data collection approaches (Cunnama *et al.*, 2016). In HICs, there is much greater reliance on reference costs rather than the ingredients cost approach. This is partly because these data are available and easily accessible, but perhaps also because they are regarded as more nationally representative than costs collected in a selection of health facilities. However, while reference costs are undoubtedly simpler to use, they may not fully capture changes in resource use; for example, an intervention may alter the quantity of staff time and materials used in the course of a ‘patient bed‐day’, and this change would not be reflected in a standardised reference cost.

Thirdly, *differing expectations and research environments* may also shape method selection. Anticipations from funders, journals and peer reviewers as to the type of methods used and the sophistication of the analysis are different between settings. For example, international donors funding studies in LLMICs often prefer DALYs to QALYs as these better facilitate comparison between settings (Santatiwongchai *et al.*, 2015). In HICs with available multi‐attribute scoring functions, official guidelines tend to require QALYs. In LLMICs, the need to collect primary cost data, which involves substantial time and resources, may reduce the scope within fixed research budgets for extensive analysis and modelling, especially where funders have not previously supported such work. Moreover, EEs from different regions are to a large extent published in separate peer‐reviewed journals with diverse methodological expectations (Pitt *et al.*, 2016). The journal *Value in Health*, for example, recently launched regional issues for Asia, Latin America, Central and Eastern Europe, Western Asia and Africa, which seems to suggest that the main issue of the journal is largely reserved for HICs. This separation discourages scholarly dialogue between researchers working in different regions and risks permitting different standards to emerge across the various issues.

Fourthly, differences in methods are also likely to stem from *different health economics capacity*. For example, in UMICs and LLMICs, modelling and sensitivity analysis were less commonly used, which may reflect a lack of technical expertise and shortage of trained health economists. Similarly, DALYs are invariably easier to apply as they do not require locally validated tools. The fact that most EE textbooks have been developed exclusively for audiences in high‐income settings – omitting entirely, for example, any guidance on the use of DALYs (Drummond *et al.*, 2005, Drummond and Mcguire, 2001) until this year (Drummond *et al*., 2015) – has hindered research capacity development in lower‐income settings and collaboration between researchers across settings. Nonetheless, the growing number of countries – particularly UMICs – producing EE guidelines is a positive sign and an opportunity to strengthen EE research.

In conclusion, some of the methodological differences we have identified rightly reflect the contextual heterogeneity across low‐income, middle‐income and high‐income countries; however, other differences appear less justified. In HICs, the widespread use of reference costs and frequent recommendation of limiting the analysis to the government health sector perspective warrant greater scrutiny. In LLMICs, particular efforts are needed for research in health‐related quality of life measurements as well as promotion of routine use of probabilistic sensitivity analysis, modelling where necessary, and statistical analysis of cost data. Moreover, routine data systems for both cost and disease surveillance would greatly facilitate LLMIC research. In all countries, further research is needed into cost‐effectiveness thresholds. Greater collaboration between researchers across settings could foster a stronger EE research community and improve methods in all settings.

## Conflict of Interest

The authors have no conflict of interest.

## Supporting information

Supporting info item
