# Supplementary material for: Comparison of Economic Evaluation Methods Across Low‐income, Middle‐income and High‐income Countries: What are the Differences and Why?
Source: Health Econ. 2016 Jan 17;25(Suppl Suppl 1):29–41. doi: 10.1002/hec.3312 (PMC5042040; doi:10.1002/hec.3312)
Supplement: Supplementary file 1 — Supporting info item [file HEC-25-29-s001.docx]

**Web annex: Papers included in cross-sectional analysis randomly selected from bibliometric database**

**Table A1: Papers from low-and lower middle-income countries**

|  | **First author (ref)** | **Country** | **Disease** | **Intervention** |
| --- | --- | --- | --- | --- |
| 1 | Puett ([1](#_ENREF_1)) | Bangladesh | Malnutrition | Management by community health workers |
| 2 | Lefevre ([2](#_ENREF_2)) | Bangladesh | Infant mortality | Neonatal care packages |
| 3 | Fottrell ([3](#_ENREF_3)) | Bangladesh | Neonatal mortality | Women's groups |
| 4 | Hsu ([4](#_ENREF_4)) | Benin | HIV | Social marketing |
| 5 | Rattray ([5](#_ENREF_5)) | Cambodia | Orthopedics | Reconstructive surgery |
| 6 | Puett ([6](#_ENREF_6)) | Chad | Malnutrition | Food assistance |
| 7 | Deboutte ([7](#_ENREF_7)) | DRC | Obstetric emergency | Caesarean section |
| 8 | Obach ([8](#_ENREF_8)) | Egypt | Hepatitis C | Immediate treatment |
| 9 | Paintain ([9](#_ENREF_9)) | Ghana | Malaria | Long-lasting insecticidal mosquito nets |
| 10 | Buttorff ([10](#_ENREF_10)) | India | Mental health | Task shifting |
| 11 | Kuriachan ([11](#_ENREF_11)) | India | Rheumatoid arthritis | Selected drug combinations |
| 12 | Pandav ([12](#_ENREF_12)) | India | Iodine deficiency disorder | Iodized oil injections and salt |
| 13 | Pho ([13](#_ENREF_13)) | India | HIV | Tuberculosis preventive therapy |
| 14 | Singh ([14](#_ENREF_14)) | India | Hospital acquired infection | Modular training in infection |
| 15 | Aggarwal ([15](#_ENREF_15)) | India | Chronic plaque psoriasis | Psoralen, ultraviolet A and sunlight |
| 16 | Clark ([16](#_ENREF_16)) | India | *Haemophilus influenza* type b | Hib vaccine |
| 17 | Gupta ([17](#_ENREF_17)) | India | *Haemophilus influenza* type b | Hib vaccine |
| 18 | Brown ([18](#_ENREF_18)) | India | Smoking | School-based smoking prevention |
| 19 | Rachapelle ([19](#_ENREF_19)) | India | Diabetic Retinopathy | Telemedicine screening |
| 20 | Suwantika ([20](#_ENREF_20)) | Indonesia | Rotavirus diarrhoea | Rotavirus vaccine |
| 21 | Ayieko ([21](#_ENREF_21)) | Kenya | Rotavirus diarrhoea | Rotavirus vaccine |
| 22 | Shade ([22](#_ENREF_22)) | Kenya | HIV/family planning | Integrated family planning and HIV services |
| 23 | Opondo ([23](#_ENREF_23)) | Kenya | Femoral shaft fractures | Surgery versus skeletal traction |
| 24 | Zurovac ([24](#_ENREF_24)) | Kenya | Malaria | Mobile phone text message to health workers |
| 25 | Rutstein ([25](#_ENREF_25)) | Malawi | HIV | Partner notification |
| 26 | Klingler ([26](#_ENREF_26)) | Mozambique | Hepatitis B | Birth dose of hepatitis B vaccine |
| 27 | Rheingans ([27](#_ENREF_27)) | Multi | Rotavirus | Rotavirus vaccine |
| 28 | Chen ([28](#_ENREF_28)) | Nicaragua | Orthopedic | Volunteer orthopedic surgical trips |
| 29 | Ezenduka ([29](#_ENREF_29)) | Nigeria | Leprosy | Increased case detection |
| 30 | Ekwunife ([30](#_ENREF_30)) | Nigeria | Hypertension | Medications |
| 31 | Adibe ([31](#_ENREF_31)) | Nigeria | Type 2 diabetes | Patient-centred care |
| 32 | Suleiman ([32](#_ENREF_32)) | Nigeria | Staphylococcal aureus infections | Three different antibiotics |
| 33 | Qureshi ([33](#_ENREF_33)) | Pakistan | Snake bite | Anti snake venom |
| 34 | Binagwaho ([34](#_ENREF_34)) | Rwanda | HIV | ART and breastfeeding |
| 35 | Clark ([35](#_ENREF_35)) | Sierra Leone | Inpatient mortality | Emergency care system |
| 36 | Senarathna ([36](#_ENREF_36)) | Sri Lanka | Paracetomol poisoning with suicidal intent | Antidotes |
| 37 | Shah ([37](#_ENREF_37)) | Uganda | Tuberculosis and HIV | Tuberculosis diagnosis |
| 38 | Sempa ([38](#_ENREF_38)) | Uganda | HIV | Early ART |
| 39 | Alfonso ([39](#_ENREF_39)) | Uganda | Obstetrics | Voucher scheme |
| 40 | Mupere ([40](#_ENREF_40)) | Uganda | Tuberculosis | Active case finding |
| 41 | Alistar ([41](#_ENREF_41)) | Ukraine | HIV in injecting drug users | Pre-Exposure Prophylaxis with ART |
| 42 | Moon ([42](#_ENREF_42)) | Vietnam | Cleft lip and cleft palate | International volunteer surgery |
| 43 | Tran ([43](#_ENREF_43)) | Vietnam | Injection-driven HIV | Integrating methadone maintenance and ART |
| 44 | Tu ([44](#_ENREF_44)) | Vietnam | Hepatitis B | Hepatitis B vaccine |
| 45 | Nichols ([45](#_ENREF_45)) | Zambia | HIV | Pre-Exposure Prophylaxis |
| 46 | Marseille ([46](#_ENREF_46)) | Zambia | HIV | ART as currently done |
| 47 | Griffiths ([47](#_ENREF_47)) | Zambia | Eye health | Cataract surgery and glasses |
| 48 | Ciaranello ([48](#_ENREF_48)) | Zimbabwe | HIV | Prevention of mother-to-child transmission |
| 49 | Miller ([49](#_ENREF_49)) | Zimbabwe | HIV | School Support for Orphan Girls |
| 50 | Ndeffo Mbah ([50](#_ENREF_50)) | Zimbabwe | Schistosoma haematobium and HIV | Clean water, sanitation and health education |

**Table A2: Papers from upper middle-income countries**

|  | **First author (ref)** | **Country** | **Disease** | **Intervention** |
| --- | --- | --- | --- | --- |
| 1 | Elgart ([51](#_ENREF_51)) | Argentina | Type 2 diabetes | Saxagliptin |
| 2 | Bertoldi ([52](#_ENREF_52)) | Brazil | Heart failure | Cardiac resynchronization therapy |
| 3 | Louzada Maldonado ([53](#_ENREF_53)) | Brazil | Fertility | Gonadotropin-releasing hormone |
| 4 | Bahia ([54](#_ENREF_54)) | Brazil | Prostatic hyperplasia | Medical treatment |
| 5 | Itria ([55](#_ENREF_55)) | Brazil | Meningococcal C | Vaccination |
| 6 | Machado ([56](#_ENREF_56)) | Brazil | Breast cancer | Lapatinib |
| 7 | Kamusheva ([57](#_ENREF_57)) | Bulgaria | Parkinsons disease | Intestinal gel Levodopa + Carbidopa |
| 8 | Hutton ([58](#_ENREF_58)) | China | Hepatitis B | Hepatitis B vaccine catch-up |
| 9 | Tobe ([59](#_ENREF_59)) | China | Hearing loss | Neonatal Hearing Screening Program |
| 10 | De Steur ([60](#_ENREF_60)) | China | Micronutrient malnutrition | Multi-biofortified rice |
| 11 | Zou ([61](#_ENREF_61)) | China | Tuberculosis | Tuberculosis control program |
| 12 | Pan ([62](#_ENREF_62)) | China | Hepatitis A | Vaccination |
| 13 | Ma ([63](#_ENREF_63)) | China | Retinal detachment | rhegmatogenous surgery |
| 14 | Lu ([64](#_ENREF_64)) | China | Hepatitis B | Early detection and treatment |
| 15 | Chen ([65](#_ENREF_65)) | China | Allergic rhinitis with asthma | Immunotherapy |
| 16 | Chen ([66](#_ENREF_66)) | China | Infertility | In vitro fertilization |
| 17 | Wu ([67](#_ENREF_67)) | China | Cardiovasular disease | Receptor blockers |
| 18 | Wu ([68](#_ENREF_68)) | China | Gastric cancer | Trastuzumab and chemotherapy |
| 19 | Zhang ([69](#_ENREF_69)) | China | Diabetes | Screening |
| 20 | Zhang ([70](#_ENREF_70)) | China | HIV and drug addiction | Methadone maintenance treatment |
| 21 | Zhu ([71](#_ENREF_71)) | China | Schistosomiasis | Comprehensive control |
| 22 | Guillermo ([72](#_ENREF_72)) | Columbia | Chronic obstructive pulmonary disease | Long-acting-beta2-agonist |
| 23 | Aponte-Gonzalez ([73](#_ENREF_73)) | Columbia | Human papilloma virus | Vaccination |
| 24 | Penaranda ([74](#_ENREF_74)) | Columbia | Hearing loss | Cochlear implant |
| 25 | Rodríguez-Martínez ([75](#_ENREF_75)) | Columbia | Asthma | Inhaled corticosteroids |
| 26 | Romero ([76](#_ENREF_76)) | Columbia | Leukemia | Nilotinib, dasatinib and imatinib |
| 27 | Nagy ([77](#_ENREF_77)) | Hungary | Uterine fibroids | Ulipristal acetate tablets |
| 28 | Gharibnaseri ([78](#_ENREF_78)) | Iran | Epilepsy | New antiepileptic drugs |
| 29 | Keshavarz ([79](#_ENREF_79)) | Iran | Diabetic Peripheral Neuropathy | Pregabalin |
| 30 | Bastani ([80](#_ENREF_80)) | Iran | Breast cancer | Adjuvant therapies |
| 31 | Hatam ([81](#_ENREF_81)) | Iran | Rabies | Post exposure prophylaxis |
| 32 | Selvarajah ([82](#_ENREF_82)) | Malaysia | Cardiovascular disease | Risk screening |
| 33 | Cortes-Sanabria ([83](#_ENREF_83)) | Mexico | Kidney disease | Peritoneal dialysis |
| 34 | Salinas-Escudero ([84](#_ENREF_84)) | Mexico | Hemophilia A | Prophylaxis vs. "on demand" |
| 35 | Salinas-Escudero ([85](#_ENREF_85)) | Mexico | Rheumatoid arthritis | Etanercept compared to other biologic therapies |
| 36 | Gutierrez-Castrello ([86](#_ENREF_86)) | Mexico | Diarrhoea | Preventive probiotics |
| 37 | Mihajlovic ([87](#_ENREF_87)) | Serbia | Kidney cancer | Everolimus |
| 38 | Kostic ([88](#_ENREF_88)) | Serbia | Rheumatoid arthritis | DMARDs alone or in combination with tocilizumab |
| 39 | Alistar ([89](#_ENREF_89)) | South Africa | HIV | ART and pre-exposure prophylaxis |
| 40 | Jarvis ([90](#_ENREF_90)) | South Africa | HIV-associated cryptococcal meningitis | Cryptococcal antigen screening |
| 41 | Mandalakas ([91](#_ENREF_91)) | South Africa | Tuberculosis | Screening of children |
| 42 | Klein ([92](#_ENREF_92)) | South Africa | AIDS | ART |
| 43 | Terris-Prestholt ([93](#_ENREF_93)) | South Africa | HIV | Tenofovir gel |
| 44 | Dye ([94](#_ENREF_94)) | South Africa | Tuberculosis | BCG vaccine |
| 45 | Vergout ([95](#_ENREF_95)) | South Africa | Various childhood diseases | Integrated vaccination campaign |
| 46 | Rattanavipapong ([96](#_ENREF_96)) | Thailand | Epilepsy and back pain | Genetic screening for adverse drug reactions |
| 47 | Khiaocharoen ([97](#_ENREF_97)) | Thailand | Stroke | Rehabilitation services |
| 48 | Kulpeng ([98](#_ENREF_98)) | Thailand | Pneumococcal | Pneumococcal conjugate vaccine |
| 49 | Muangman ([99](#_ENREF_99)) | Thailand | Pulmonary embolism | Diagnostics |
| 50 | Prukkanone ([100](#_ENREF_100)) | Thailand | Depression | Antidepressants and cognitive behavioural therapy |

**Table S3: Papers from high-income countries**

|  | **First author (ref)** | **Country** | **Disease** | **Intervention** |
| --- | --- | --- | --- | --- |
| 1 | Tan ([101](#_ENREF_101)) | Australia | Stroke | Thrombolysis within 4.5 hours |
| 2 | Hettle ([102](#_ENREF_102)) | Belgium, UK | Chronic obstructive pulmonary disease | Tiotropium |
| 3 | Chui ([103](#_ENREF_103)) | Canada | Trauma | Epoetin alfa |
| 4 | Kwon ([104](#_ENREF_104)) | Canada | Ovarian cancer | Prophylactic Salpingectomy and Delayed Oophorectomy |
| 5 | Mauskopf ([105](#_ENREF_105)) | Canada | HIV | Combination therapy with etravirine |
| 6 | Singh ([106](#_ENREF_106)) | Canada | Stroke | Atrial appendage occlusion, dabigatran, and warfarin |
| 7 | Zurawska ([107](#_ENREF_107)) | Canada | Lymphoma | Hepatitis B screening before chemotherapy |
| 8 | Guest ([108](#_ENREF_108)) | France, Germany, UK | Chronic wounds | Polyheal |
| 9 | Hartz ([109](#_ENREF_109)) | Germany | Alzheimer | Donepezil |
| 10 | Athanasakis ([110](#_ENREF_110)) | Greece | Hypertension | Hypertension drugs |
| 11 | Liberato ([111](#_ENREF_111)) | Italy | Head and neck cancer | Docetaxel |
| 12 | Liguori ([112](#_ENREF_112)) | Italy | Pneumococcal | Pneumococcal conjugate vaccine |
| 13 | Ruggeri ([113](#_ENREF_113)) | Italy | Colorectal cancer | Screening |
| 14 | Iannazzo ([114](#_ENREF_114)) | Italy, Spain, Portugal, Czech Republic, Switzerland | Kidney disease | Cinacalcet |
| 15 | Lokkerbol ([115](#_ENREF_115)) | Netherlands | Depression | Telemedicine |
| 16 | Meijboom ([116](#_ENREF_116)) | Netherlands | Respiratory syncytial virus | Vaccine |
| 17 | Mohseninejad ([117](#_ENREF_117)) | Netherlands | Coeliac Disease in irritable bowel syndrome | Screening |
| 18 | van Kessel ([118](#_ENREF_118)) | Netherlands | Bladder cancer | FGFR3 mutation analysis of voided urine |
| 19 | van Steensel ([119](#_ENREF_119)) | Netherlands | Anxiety in autistic children | Cognitive behavioural therapy |
| 20 | de Bock ([120](#_ENREF_120)) | Netherlands, UK, USA | Breast cancer | Screening |
| 21 | Boberg ([121](#_ENREF_121)) | Norway | Cirrhosis | Ursodeoxycholic acid |
| 22 | Oppong ([122](#_ENREF_122)) | Norway, Sweden | Lower respiratory tract infections | Rapid point of care test |
| 23 | Carrera-Hueso ([123](#_ENREF_123)) | Spain | Ventilator-associated pneumonia | Doripenem or imipenem |
| 24 | Blasco ([124](#_ENREF_124)) | Spain | HIV | ART |
| 25 | Diaz ([125](#_ENREF_125)) | Spain | Emergency complications | Prothrombin complex concentrate |
| 26 | Rubio-Valera ([126](#_ENREF_126)) | Spain | Depression | Community Pharmacist adherence counselling |
| 27 | Ohsfeldt ([127](#_ENREF_127)) | Sweden | Cardiovascular | Rosuvastatin |
| 28 | Wu ([128](#_ENREF_128)) | Taiwan | Pneumococcal | Pneumococcal conjugate vaccine |
| 29 | Ara ([129](#_ENREF_129)) | UK | Obesity | Obesity drugs |
| 30 | Brown ([130](#_ENREF_130)) | UK | Lung cancer | Chemotherapy |
| 31 | Carroll ([131](#_ENREF_131)) | UK | Varicose veins | Three minimally invasive techniques |
| 32 | Ewer ([132](#_ENREF_132)) | UK | Congenital heart defects in new-borns | Screening |
| 33 | Mavranezouli ([133](#_ENREF_133)) | UK | Anxiety | Pharmacological treatments |
| 34 | Miners ([134](#_ENREF_134)) | UK | Obesity | E-learning |
| 35 | O'Cathail ([135](#_ENREF_135)) | UK | Gynaecological cancers | Intravenous vs. oral dexamethasone |
| 36 | Svedbom ([136](#_ENREF_136)) | UK | Acute osteoporotic vertebral compression fracture | Balloon kyphoplasty vs. vertebroplasty vs. nonsurgical |
| 37 | Allen ([137](#_ENREF_137)) | USA | Cardiovascular | Nurse vs. community health worker |
| 38 | Clark ([138](#_ENREF_138)) | USA | Well-being in elderly | Lifestyle intervention |
| 39 | Folse ([139](#_ENREF_139)) | USA | Breast cancer | Genetic test |
| 40 | Goulart ([140](#_ENREF_140)) | USA | Lung cancer | Screening |
| 41 | Jutkowitz ([141](#_ENREF_141)) | USA | Age related mortality | Home-based intervention for elderly |
| 42 | Kacker ([142](#_ENREF_142)) | USA | Sickle cell | Screening of donor blood |
| 43 | Kamel ([143](#_ENREF_143)) | USA | Stroke | Apixaban vs warfarin |
| 44 | Mather ([144](#_ENREF_144)) | USA | Knee arthritis | Knee surgery |
| 45 | Najafzadeh ([145](#_ENREF_145)) | USA | Thyroid cancer | Molecular diagnostic test |
| 46 | Reese ([146](#_ENREF_146)) | USA | Cardiovascular and stroke | Genotype-guided selection of antiplatelet therapy |
| 47 | Schackman ([147](#_ENREF_147)) | USA | Drug addiction | Buprenorphine-Naloxone |
| 48 | Stein ([148](#_ENREF_148)) | USA | Glaucoma | Medications vs laser trabeculoplasty |
| 49 | Teresi ([149](#_ENREF_149)) | USA | Elderly falls and QOL | Education in nursing homes |
| 50 | Wang ([150](#_ENREF_150)) | USA | Obesity | Bariatric surgery |

**References**

1. Puett C, Sadler K, Alderman H, Coates J, Fiedler JL, Myatt M. Cost-effectiveness of the community-based management of severe acute malnutrition by community health workers in southern Bangladesh. Health policy and planning. 2013;28(4):386-99.

2. LeFevre AE, Shillcutt SD, Waters HR, Haider S, El Arifeen S, Mannan I, et al. Economic evaluation of neonatal care packages in a cluster-randomized controlled trial in Sylhet, Bangladesh. Bulletin of the World Health Organization. 2013;91(10):736-45.

3. Fottrell E, Azad K, Kuddus A, Younes L, Shaha S, Nahar T, et al. The effect of increased coverage of participatory women's groups on neonatal mortality in Bangladesh: A cluster randomized trial. JAMA pediatrics. 2013;167(9):816-25.

4. Hsu J, Zinsou C, Parkhurst J, N'Dour M, Foyet L, Mueller DH. Comparative costs and cost-effectiveness of behavioural interventions as part of HIV prevention strategies. Health policy and planning. 2013;28(1):20-9.

5. Rattray KW, Harrop, T. C., Aird, J., Tam, T., Beveridge, M., Gollogly, J. G. The cost effectiveness of reconstructive surgery in Cambodia. Asian Biomedicine. 2013;7(3):319-324.

6. Puett C, Salpeteur C, Lacroix E, Houngbe F, Ait-Aissa M, Israel AD. Protecting child health and nutrition status with ready-to-use food in addition to food assistance in urban Chad: a cost-effectiveness analysis. Cost effectiveness and resource allocation : C/E. 2013;11(1):27.

7. Deboutte D, O'Dempsey T, Mann G, Faragher B. Cost-effectiveness of caesarean sections in a post-conflict environment: a case study of Bunia, Democratic Republic of the Congo. Disasters. 2013;37 Suppl 1:S105-20.

8. Obach D, Deuffic-Burban S, Esmat G, Anwar WA, Dewedar S, Canva V, et al. Effectiveness and cost-effectiveness of immediate versus delayed treatment of hepatitis C virus-infected patients in a country with limited resources: the case of Egypt. Clinical infectious diseases : an official publication of the Infectious Diseases Society of America. 2014;58(8):1064-71.

9. Smith Paintain L, Awini E, Addei S, Kukula V, Nikoi C, Sarpong D, et al. Evaluation of a universal long-lasting insecticidal net (LLIN) distribution campaign in Ghana: cost effectiveness of distribution and hang-up activities. Malaria journal. 2014;13:71.

10. Buttorff C, Hock RS, Weiss HA, Naik S, Araya R, Kirkwood BR, et al. Economic evaluation of a task-shifting intervention for common mental disorders in India. Bulletin of the World Health Organization. 2012;90(11):813-21.

11. Kuriachan MA, Revikumar, K.G., Jolly, A. Comparison of treatment outcome in rheumatoid arthritis patients treated with single and two DMARDs in combination with corticosteroids. International Journal of Drug Development and Research. 2012;4(3):228-35.

12. Pandav CS. Economic evaluation of iodine deficiency disorder control program in Sikkim: a cost effectiveness study. Indian journal of public health. 2012;56(1):37-43.

13. Pho MT, Swaminathan S, Kumarasamy N, Losina E, Ponnuraja C, Uhler LM, et al. The cost-effectiveness of tuberculosis preventive therapy for HIV-infected individuals in southern India: a trial-based analysis. PloS one. 2012;7(4):e36001.

14. Singh S, Kumar RK, Sundaram KR, Kanjilal B, Nair P. Improving outcomes and reducing costs by modular training in infection control in a resource-limited setting. International journal for quality in health care : journal of the International Society for Quality in Health Care / ISQua. 2012;24(6):641-8.

15. Aggarwal K, Khandpur S, Khanna N, Sharma VK, Pandav CS. Comparison of clinical and cost-effectiveness of psoralen + ultraviolet A versus psoralen + sunlight in the treatment of chronic plaque psoriasis in a developing economy. International journal of dermatology. 2013;52(4):478-85.

16. Clark AD, Griffiths UK, Abbas SS, Rao KD, Privor-Dumm L, Hajjeh R, et al. Impact and cost-effectiveness of Haemophilus influenzae type b conjugate vaccination in India. The Journal of pediatrics. 2013;163(1 Suppl):S60-72.

17. Gupta M, Prinja S, Kumar R, Kaur M. Cost-effectiveness of Haemophilus influenzae type b (Hib) vaccine introduction in the universal immunization schedule in Haryana State, India. Health policy and planning. 2013;28(1):51-61.

18. Brown HS, 3rd, Stigler M, Perry C, Dhavan P, Arora M, Reddy KS. The cost-effectiveness of a school-based smoking prevention program in India. Health promotion international. 2013;28(2):178-86.

19. Rachapelle S, Legood R, Alavi Y, Lindfield R, Sharma T, Kuper H, et al. The cost-utility of telemedicine to screen for diabetic retinopathy in India. Ophthalmology. 2013;120(3):566-73.

20. Suwantika AA, Tu HA, Postma MJ. Cost-effectiveness of rotavirus immunization in Indonesia: taking breastfeeding patterns into account. Vaccine. 2013;31(32):3300-7.

21. Ayieko P, Griffiths UK, Ndiritu M, Moisi J, Mugoya IK, Kamau T, et al. Assessment of health benefits and cost-effectiveness of 10-valent and 13-valent pneumococcal conjugate vaccination in Kenyan children. PloS one. 2013;8(6):e67324.

22. Shade SB, Kevany S, Onono M, Ochieng G, Steinfeld RL, Grossman D, et al. Cost, cost-efficiency and cost-effectiveness of integrated family planning and HIV services. Aids. 2013;27 Suppl 1:S87-92.

23. Opondo E WPMA. Cost effectiveness of using surgery versus skeletal traction in management of femoral shaft fractures at Thika level 5 hospital, Kenya. Pan African Medical Journal. 2013;15(None):42.

24. Zurovac D, Larson BA, Sudoi RK, Snow RW. Costs and cost-effectiveness of a mobile phone text-message reminder programmes to improve health workers' adherence to malaria guidelines in Kenya. PloS one. 2012;7(12):e52045.

25. Rutstein SE, Brown LB, Biddle AK, Wheeler SB, Kamanga G, Mmodzi P, et al. Cost-effectiveness of provider-based HIV partner notification in urban Malawi. Health policy and planning. 2014;29(1):115-26.

26. Klingler C, Thoumi AI, Mrithinjayam VS. Cost-effectiveness analysis of an additional birth dose of Hepatitis B vaccine to prevent perinatal transmission in a medical setting in Mozambique. Vaccine. 2012;31(1):252-9.

27. Rheingans R, Atherly D, Anderson J. Distributional impact of rotavirus vaccination in 25 GAVI countries: estimating disparities in benefits and cost-effectiveness. Vaccine. 2012;30 Suppl 1:A15-23.

28. Chen AT, Pedtke A, Kobs JK, Edwards GS, Jr., Coughlin RR, Gosselin RA. Volunteer orthopedic surgical trips in Nicaragua: a cost-effectiveness evaluation. World journal of surgery. 2012;36(12):2802-8.

29. Ezenduka C, Post E, John S, Suraj A, Namadi A, Onwujekwe O. Cost-effectiveness analysis of three leprosy case detection methods in Northern Nigeria. PLoS neglected tropical diseases. 2012;6(9):e1818.

30. Ekwunife OI, Okafor CE, Ezenduka CC, Udeogaranya PO. Cost-utility analysis of antihypertensive medications in Nigeria: a decision analysis. Cost effectiveness and resource allocation : C/E. 2013;11(1):2.

31. Adibe Mo ACNUCV. Cost-utility analysis of pharmaceutical care intervention versus usual care in management of Nigerian patients with type 2 diabetes. Value in Health Regional Issues. 2013;2(2):189-198.

32. Suleiman IA, Bamiro BS, Tayo F. Cost effectiveness of three drugs for the treatment of S. aureus infections in Nigeria. International journal of clinical pharmacy. 2012;34(5):739-45.

33. Qureshi H, Alam SE, Mustufa MA, Nomani NK, Asnani JL, Sharif M. Comparative cost and efficacy trial of Pakistani versus Indian anti snake venom. JPMA The Journal of the Pakistan Medical Association. 2013;63(9):1129-32.

34. Binagwaho A, Pegurri E, Drobac PC, Mugwaneza P, Stulac SN, Wagner CM, et al. Prevention of mother-to-child transmission of HIV: cost-effectiveness of antiretroviral regimens and feeding options in Rwanda. PloS one. 2013;8(2):e54180.

35. Clark M, Spry E, Daoh K, Baion D, Skordis-Worrall J. Reductions in inpatient mortality following interventions to improve emergency hospital care in Freetown, Sierra Leone. PloS one. 2012;7(9):e41458.

36. Senarathna SM, Sri Ranganathan S, Buckley N, Fernandopulle R. A cost effectiveness analysis of the preferred antidotes for acute paracetamol poisoning patients in Sri Lanka. BMC clinical pharmacology. 2012;12:6.

37. Shah M, Dowdy D, Joloba M, Ssengooba W, Manabe YC, Ellner J, et al. Cost-effectiveness of novel algorithms for rapid diagnosis of tuberculosis in HIV-infected individuals in Uganda. Aids. 2013;27(18):2883-92.

38. Sempa J, Ssennono M, Kuznik A, Lamorde M, Sowinski S, Semeere A, et al. Cost-effectiveness of early initiation of first-line combination antiretroviral therapy in Uganda. BMC public health. 2012;12:736.

39. Alfonso YN, Bishai D, Bua J, Mutebi A, Mayora C, Ekirapa-Kiracho E. Cost-effectiveness analysis of a voucher scheme combined with obstetrical quality improvements: quasi experimental results from Uganda. Health policy and planning. 2015;30(1):88-99.

40. Mupere E, Schiltz NK, Mulogo E, Katamba A, Nabbuye-Sekandi J, Singer ME. Effectiveness of active case-finding strategies in tuberculosis control in Kampala, Uganda. The international journal of tuberculosis and lung disease : the official journal of the International Union against Tuberculosis and Lung Disease. 2013;17(2):207-13.

41. Alistar SS, Owens DK, Brandeau ML. Effectiveness and cost effectiveness of oral pre-exposure prophylaxis in a portfolio of prevention programs for injection drug users in mixed HIV epidemics. PloS one. 2014;9(1):e86584.

42. Moon W, Perry, H., Baek, R.M. Is international volunteer surgery for cleft lip and cleft palate a cost-effective and justifiable intervention? A case study from East Asia. World journal of surgery. 2012;36(12):2819-30.

43. Tran BX, Nguyen LT. Impact of methadone maintenance on health utility, health care utilization and expenditure in drug users with HIV/AIDS. The International journal on drug policy. 2013;24(6):e105-10.

44. Tu HAT, de Vries, R., Woerdenbag, H.J., Li, S.C., Le, H.H., van Hulst, M., Postma, M.J. Cost-Effectiveness Analysis of Hepatitis B Immunization in Vietnam: Application of Cost-Effectiveness Affordability Curves in Health Care Decision Making. Value in Health Regional Issues. 2012;1(1):7-14.

45. Nichols BE, Boucher CA, van Dijk JH, Thuma PE, Nouwen JL, Baltussen R, et al. Cost-effectiveness of pre-exposure prophylaxis (PrEP) in preventing HIV-1 infections in rural Zambia: a modeling study. PloS one. 2013;8(3):e59549.

46. Marseille E, Giganti MJ, Mwango A, Chisembele-Taylor A, Mulenga L, Over M, et al. Taking ART to scale: determinants of the cost and cost-effectiveness of antiretroviral therapy in 45 clinical sites in Zambia. PloS one. 2012;7(12):e51993.

47. Griffiths UK, Bozzani FM, Gheorghe A, Mwenge L, Gilbert C. Cost-effectiveness of eye care services in Zambia. Cost effectiveness and resource allocation : C/E. 2014;12(1):6.

48. Ciaranello AL, Perez F, Engelsmann B, Walensky RP, Mushavi A, Rusibamayila A, et al. Cost-effectiveness of World Health Organization 2010 guidelines for prevention of mother-to-child HIV transmission in Zimbabwe. Clinical infectious diseases : an official publication of the Infectious Diseases Society of America. 2013;56(3):430-46.

49. Miller T, Hallfors D, Cho H, Luseno W, Waehrer G. Cost-effectiveness of school support for orphan girls to prevent HIV infection in Zimbabwe. Prevention science : the official journal of the Society for Prevention Research. 2013;14(5):503-12.

50. Ndeffo Mbah ML, Kjetland EF, Atkins KE, Poolman EM, Orenstein EW, Meyers LA, et al. Cost-effectiveness of a community-based intervention for reducing the transmission of Schistosoma haematobium and HIV in Africa. Proceedings of the National Academy of Sciences of the United States of America. 2013;110(19):7952-7.

51. Elgart JF, Caporale JE, Gonzalez L, Aiello E, Waschbusch M, Gagliardino JJ. Treatment of type 2 diabetes with saxagliptin: a pharmacoeconomic evaluation in Argentina. Health economics review. 2013;3(1):11.

52. Bertoldi EG, Rohde LE, Zimerman LI, Pimentel M, Polanczyk CA. Cost-effectiveness of cardiac resynchronization therapy in patients with heart failure: the perspective of a middle-income country's public health system. International journal of cardiology. 2013;163(3):309-15.

53. Maldonado LG, Franco JG, Jr., Setti AS, Iaconelli A, Jr., Borges E, Jr. Cost-effectiveness comparison between pituitary down-regulation with a gonadotropin-releasing hormone agonist short regimen on alternate days and an antagonist protocol for assisted fertilization treatments. Fertility and sterility. 2013;99(6):1615-22.

54. Bahia LR, Araujo DV, Pepe C, Trindade M, Camargo CM, Javaroni V. Cost-effectiveness analysis of medical treatment of benign prostatic hyperplasia in the Brazilian public health system. International braz j urol : official journal of the Brazilian Society of Urology. 2012;38(5):595-605.

55. Itria A, Novaes HMD, Soárez PCd, Nobrega LdAL, Sartori AMC. A importancia dos metodos de custeio e valoracão nas avaliacoes economicas em saude: repercussoes sobre os resultados de avaliacão da vacina antimeningococica C. Physis: Revista de Saude Coletiva. 2012;22(2):641-658.

56. Machado M, Einarson TR. Lapatinib in patients with metastatic breast cancer following initial treatment with trastuzumab: an economic analysis from the Brazilian public health care perspective. Breast cancer. 2012;4:173-82.

57. Kamusheva M.S GNPGI. Intestinal gel Levodopa + Carbidopa in Parkinson's patients with frequent and prolonged akinesia - an economic evaluation. International Journal of Pharmaceutical Sciences Review and Research. 2013;22(1):244-246.

58. Hutton DW, Brandeau ML. Too much of a good thing? When to stop catch-up vaccination. Medical decision making : an international journal of the Society for Medical Decision Making. 2013;33(7):920-36.

59. Tobe RG, Mori R, Huang L, Xu L, Han D, Shibuya K. Cost-effectiveness analysis of a national neonatal hearing screening program in China: conditions for the scale-up. PloS one. 2013;8(1):e51990.

60. De Steur H, Gellynck X, Blancquaert D, Lambert W, Van Der Straeten D, Qaim M. Potential impact and cost-effectiveness of multi-biofortified rice in China. New biotechnology. 2012;29(3):432-42.

61. Zou G WXWSYJWJLSYHCJTGMJ. Incremental cost-effectiveness of improving treatment results among migrant tuberculosis patients in Shanghai. International Journal of Tuberculosis and Lung Disease. 2013;17(8):1056-1064.

62. Pan XJ, Feng YM, Zhuang GH. [Cost-utility analysis on universal childhood hepatitis A vaccination in regions with different anti-HAV prevalence rates of China]. Zhonghua liu xing bing xue za zhi = Zhonghua liuxingbingxue zazhi. 2012;33(8):862-6.

63. Ma Y, Ying X, Zou H, Xu X, Liu H, Bai L, et al. Cost-utility Analysis of Rhegmatogenous Retinal Detachment Surgery in Shanghai, China. Ophthalmic epidemiology. 2015;22(1):13-9.

64. Lu SQ, McGhee SM, Xie X, Cheng J, Fielding R. Economic evaluation of universal newborn hepatitis B vaccination in China. Vaccine. 2013;31(14):1864-9.

65. Chen J, Xiang J, Wang Y, Shi Q, Tan H, Kong W. [Health economics analysis of specific immunotherapy in allergic rhinitis accompanied with asthma]. Lin chuang er bi yan hou tou jing wai ke za zhi = Journal of clinical otorhinolaryngology, head, and neck surgery. 2013;27(17):925-8.

66. Chen X, Geng L, Li H. [Clinical outcomes and economic analysis of two ovulation induction protocols in patients undergoing repeated IVF/ICSI cycles]. Nan fang yi ke da xue xue bao = Journal of Southern Medical University. 2014;34(4):563-7.

67. Wu Y, Zhou, Q., Xuan, J., Li, M., Zelt, S., Huang, Y., Yin, H., Huang, M. A cost-effectiveness analysis between amlodipine and angiotensin II receptor blockers in stroke and myocardial infarction prevention among hypertension patients in China. Value in Health Regional Issues 2013;2(1):75-80.

68. Wu B, Ye M, Chen H, Shen JF. Costs of trastuzumab in combination with chemotherapy for HER2-positive advanced gastric or gastroesophageal junction cancer: an economic evaluation in the Chinese context. Clinical therapeutics. 2012;34(2):468-79.

69. Zhang Y, Sun J, Pang Z, Gao W, Sintonen H, Kapur A, et al. Evaluation of two screening methods for undiagnosed diabetes in China: an cost-effectiveness study. Primary care diabetes. 2013;7(4):275-82.

70. Zhang G, Hu YY, Xue H, Shan D, Sun Y, Yang YC, et al. [Health economic evaluation for the extension clinics of methadone maintenance treatment]. Zhonghua yu fang yi xue za zhi [Chinese journal of preventive medicine]. 2013;47(11):996-1000.

71. Zhu JY, Wang CX, Wang CF, Wang SZ, Lu K, Hu XJ, et al. [Cost-effectiveness of schistosomiasis comprehensive control in Lushan County from 2007 to 2012]. Zhongguo xue xi chong bing fang zhi za zhi = Chinese journal of schistosomiasis control. 2013;25(5):513-5.

72. Guillermo Ariza J TPOMGMLKMAYFGL. The cost-effectiveness and budget impact of introducing indacaterol into the Colombian health system. Value in Health Regional Issues. 2012;1(2):165-171.

73. Aponte-Gonzalez J, Fajardo-Bernal L, Diaz J, Eslava-Schmalbach J, Gamboa O, Hay JW. Cost-effectiveness analysis of the bivalent and quadrivalent human papillomavirus vaccines from a societal perspective in Colombia. PloS one. 2013;8(11):e80639.

74. Peñaranda A, Mendieta, J.C., Perdomo, J.A., Aparicio, M.L., Marín, L.M., García, J.M., Barón, C. Economic benefits of the cochlear implant for treating profound sensorineural hearing loss [Spanish]. Revista Panamericana de Salud Publica. 2012;31(4):325-331.

75. Rodriguez-Martinez CE, Sossa-Briceno MP, Castro-Rodriguez JA. Cost-utility analysis of the inhaled steroids available in a developing country for the management of pediatric patients with persistent asthma. The Journal of asthma : official journal of the Association for the Care of Asthma. 2013;50(4):410-8.

76. Romero M, Chavez D, De los Rios M, Alvis-Guzman N. Cost-effectiveness of nilotinib, dasatinib and imatinib as first-line treatment for chronic myeloid leukemia in Colombia, 2012. Biomedica. 2014;34(1):48-59.

77. Nagy B, Timar G, Jozwiak-Hagymasy J, Kovacs G, Meresz G, Vamossy I, et al. The cost-effectiveness of ulipristal acetate tablets in treating patients with moderate to severe symptoms of uterine fibroids. European journal of obstetrics, gynecology, and reproductive biology. 2014;175:75-81.

78. Gharibnaseri Z, Kebriaeezadeh A, Nikfar S, Zamani G, Abdollahiasl A. Cost-effectiveness of adding-on new antiepileptic drugs to conventional regimens in controlling intractable seizures in children. Daru : journal of Faculty of Pharmacy, Tehran University of Medical Sciences. 2012;20(1):17.

79. Keshavarz K H-MA, Gharibnaseri Z, Nikfar S, Kebriaeezadeh A, Abdollahi M. The Effectiveness and Cost-effectiveness of Pregabalin in the Treatment of Diabetic Peripheral Neuropathy: A Systematic Review and Economic Model. Archives of Medical Science 2013;9(6):961-7.

80. Bastani P, Kiadaliri AA. Cost-utility analysis of adjuvant therapies for breast cancer in Iran. International journal of technology assessment in health care. 2012;28(2):110-4.

81. Hatam N, Esmaelzade F, Mirahmadizadeh A, Keshavarz K, Rajabi A, Afsar Kazerooni P, et al. Cost-effectiveness of rabies post exposure prophylaxis in iran. Journal of research in health sciences. 2014;14(2):122-7.

82. Selvarajah S HJKGGHTBACCKBML. Identification of effective screening strategies for cardiovascular disease prevention in a developing country: using cardiovascular risk-estimation and risk-reduction tools for policy recommendations. BMC Cardiovascular Disorders. 2013;13(10):None "AB - Background Recent increases in cardiovascular risk-factor prevalences have led to new national policy recommendations of universal screening for primary prevention of cardiovascular disease in Malaysia. This study assessed whether the current national policy recommendation of universal screening was optimal, by comparing the effectiveness and impact of various cardiovascular screening strategies. Methods Data from a national population based survey of 24 270 participants aged 30 to 74 was used. Five screening strategies were modelled for the overall population and by gender; universal and targeted screening (four age cut-off points). Screening strategies were assessed based on the ability to detect high cardiovascular risk populations (effectiveness), incremental effectiveness, impact on cardiovascular event prevention and cost of screening. Results 26.7% (95% confidence limits 25.7, 27.7) were at high cardiovascular risk, men 34.7% (33.6, 35.8) and women 18.9% (17.8, 20). Universal screening identified all those at high-risk and resulted in one high-risk individual detected for every 3.7 people screened, with an estimated cost of USD60. However, universal screening resulted in screening an additional 7169 persons, with an incremental cost of USD115,033 for detection of one additional high-risk individual in comparison to targeted screening of those aged ≥35 years. The cost, incremental cost and impact of detection of high-risk individuals were more for women than men for all screening strategies. The impact of screening women aged ≥45 years was similar to universal screening in men. Conclusions Targeted gender- and age-specific screening strategies would ensure more optimal utilisation of scarce resources compared to the current policy recommendations of universal screening.".

83. Cortes-Sanabria L, Paredes-Cesena CA, Herrera-Llamas RM, Cruz-Bueno Y, Soto-Molina H, Pazarin L, et al. Comparison of cost-utility between automated peritoneal dialysis and continuous ambulatory peritoneal dialysis. Archives of medical research. 2013;44(8):655-61.

84. Salinas-Escudero G G-SRMRKC-VMFM-OE. Cost-effectiveness analysis of prophylaxis vs. 'on demand' approach in the management in children with hemophilia A in Mexico [Análisis del costo y la efectividad de los esquemas de administracion de factores de coagulacion para el manejo de ninos con hemofilia A en Mexico]. Boletin Medico del Hospital Infantil de Mexico. 2013;70(4):290-298.

85. Salinas-Escudero G, Vargas-Valencia J, Garcia-Garcia EG, Muncino-Ortega E, Galindo-Suarez RM. [Cost-effectiveness analysis of etanercept compared with other biologic therapies in the treatment of rheumatoid arthritis]. Revista medica del Instituto Mexicano del Seguro Social. 2013;51(5):514-21.

86. Gutierrez-Castrellon P, Lopez-Velazquez G, Diaz-Garcia L, Jimenez-Gutierrez C, Mancilla-Ramirez J, Estevez-Jimenez J, et al. Diarrhea in preschool children and Lactobacillus reuteri: a randomized controlled trial. Pediatrics. 2014;133(4):e904-9.

87. Mihajlovic J, Pechlivanoglou P, Sabo A, Tomic Z, Postma MJ. Cost-effectiveness of everolimus for second-line treatment of metastatic renal cell carcinoma in Serbia. Clinical therapeutics. 2013;35(12):1909-22.

88. Kostic M, Jovanovic S, Tomovic M, Milenkovic MP, Jankovic SM. Cost-effectiveness analysis of tocilizumab in combination with methotrexate for rheumatoid arthritis: a Markov model based on data from Serbia, country in socioeconomic transition. Vojnosanitetski pregled Military-medical and pharmaceutical review. 2014;71(2):144-8.

89. Alistar SS, Grant PM, Bendavid E. Comparative effectiveness and cost-effectiveness of antiretroviral therapy and pre-exposure prophylaxis for HIV prevention in South Africa. BMC medicine. 2014;12:46.

90. Jarvis JN, Harrison TS, Lawn SD, Meintjes G, Wood R, Cleary S. Cost effectiveness of cryptococcal antigen screening as a strategy to prevent HIV-associated cryptococcal meningitis in South Africa. PloS one. 2013;8(7):e69288.

91. Mandalakas AM, Hesseling AC, Gie RP, Schaaf HS, Marais BJ, Sinanovic E. Modelling the cost-effectiveness of strategies to prevent tuberculosis in child contacts in a high-burden setting. Thorax. 2013;68(3):247-55.

92. Klein DJ, Bershteyn A, Eckhoff PA. Dropout and re-enrollment: implications for epidemiological projections of treatment programs. Aids. 2014;28 Suppl 1:S47-59.

93. Terris-Prestholt F, Foss AM, Cox AP, Heise L, Meyer-Rath G, Delany-Moretlwe S, et al. Cost-effectiveness of tenofovir gel in urban South Africa: model projections of HIV impact and threshold product prices. BMC infectious diseases. 2014;14:14.

94. Dye C. Making wider use of the world's most widely used vaccine: Bacille Calmette-Guerin revaccination reconsidered. Journal of the Royal Society, Interface / the Royal Society. 2013;10(87):20130365.

95. Verguet S, Jassat W, Bertram MY, Tollman SM, Murray CJ, Jamison DT, et al. Supplementary immunization activities (SIAs) in South Africa: comprehensive economic evaluation of an integrated child health delivery platform. Global health action. 2013;6:1-9.

96. Rattanavipapong W, Koopitakkajorn T, Praditsitthikorn N, Mahasirimongkol S, Teerawattananon Y. Economic evaluation of HLA-B*15:02 screening for carbamazepine-induced severe adverse drug reactions in Thailand. Epilepsia. 2013;54(9):1628-38.

97. Khiaocharoen O PSRWILTY. Economic evaluation of rehabilitation services for inpatients with stroke in Thailand: a prospective cohort study. Value in Health Regional Issues. 2012;1(1):29-35.

98. Kulpeng W, Leelahavarong P, Rattanavipapong W, Sornsrivichai V, Baggett HC, Meeyai A, et al. Cost-utility analysis of 10- and 13-valent pneumococcal conjugate vaccines: protection at what price in the Thai context? Vaccine. 2013;31(26):2839-47.

99. Muangman N, Totanarungroj K. Cost effectiveness of combined CT pulmonary angiography (CTPA) and indirect CTV in patient with intermediate to high probability for pulmonary embolism. Journal of the Medical Association of Thailand = Chotmaihet thangphaet. 2012;95(10):1321-6.

100. Prukkanone B, Vos T, Bertram M, Lim S. Cost-effectiveness analysis for antidepressants and cognitive behavioral therapy for major depression in Thailand. Value Health. 2012;15(1 Suppl):S3-8.

101. Tan Tanny SPLDSSMB. Cost-effectiveness of thrombolysis within 4.5 hours of acute ischemic stroke: experience from Australian stroke center. Stroke. 2013;44(8):2269-2274.

102. Hettle R, Wouters H, Ayres J, Gani R, Kelly S, Lion M, et al. Cost-utility analysis of tiotropium versus usual care in patients with COPD in the UK and Belgium. Respiratory medicine. 2012;106(12):1722-33.

103. Chui BK, Pannu N, Hazel M, Dong J, Tonelli M, Klarenbach SW. Economic analysis of epoetin alfa in critically ill trauma patients. The journal of trauma and acute care surgery. 2012;73(1):195-201.

104. Kwon JS, Tinker A, Pansegrau G, McAlpine J, Housty M, McCullum M, et al. Prophylactic salpingectomy and delayed oophorectomy as an alternative for BRCA mutation carriers. Obstetrics and gynecology. 2013;121(1):14-24.

105. Mauskopf J, Brogan AJ, Talbird SE, Martin S. Cost-effectiveness of combination therapy with etravirine in treatment-experienced adults with HIV-1 infection. Aids. 2012;26(3):355-64.

106. Singh S.M MAWHC. Economic evaluation of percutaneous left atrial appendage occlusion, dabigatran, and warfarin for stroke prevention in patients with nonvalvular atrial fibrillation. Circulation. 2013;127(24):2414-2423.

107. Zurawska U HLKWGBCMKMCKKFJJ. Hepatitis B virus screening before chemotherapy for lymphoma: A cost-effectiveness analysis. Journal of Clinical Oncology. 2012;30(26):3167-3173.

108. Guest J.F SEPM. Cost-effectiveness of using Polyheal compared with surgery in the management of chronic wounds with exposed bones and/or tendons due to trauma in France, Germany and the UK. International Wound Journal. 2013;None(None):None.

109. Hartz S, Getsios D, Tao S, Blume S, Maclaine G. Evaluating the cost effectiveness of donepezil in the treatment of Alzheimer's disease in Germany using discrete event simulation. BMC neurology. 2012;12:2.

110. Athanasakis K, Souliotis K, Tountas Y, Kyriopoulos J, Hatzakis A. A cost-utility analysis of hypertension treatment in Greece: assessing the impact of age, sex and smoking status, on outcomes. Journal of hypertension. 2012;30(1):227-34.

111. Liberato NL, Rognoni C, Rubrichi S, Quaglini S, Marchetti M, Gorlia T, et al. Adding docetaxel to cisplatin and fluorouracil in patients with unresectable head and neck cancer: a cost-utility analysis. Annals of oncology : official journal of the European Society for Medical Oncology / ESMO. 2012;23(7):1825-32.

112. Liguori G, Parlato A, Zamparelli AS, Belfiore P, Galle F, Di Onofrio V, et al. Adult immunization with 13-valent pneumococcal vaccine in Campania region, South Italy: an economic evaluation. Human vaccines & immunotherapeutics. 2014;10(2):492-7.

113. Ruggeri M, Coretti, S., Di Bidino, R., Marcellusi, A., Mennini, F., Cicchetti, A. Economic evaluation of a colorectal cancer screening program: Indirect evidence on longterm outcomes and economic sustainability in a regional perspective. Pharmacoeconomics - Italian Research Articles. 2012;14(2):101-20.

114. Iannazzo S, Carsi M, Chiroli S. A cost-utility analysis of cinacalcet in secondary hyperparathyroidism in five European countries. Applied health economics and health policy. 2012;10(2):127-38.

115. Lokkerbol J, Adema D, Cuijpers P, Reynolds CF, 3rd, Schulz R, Weehuizen R, et al. Improving the cost-effectiveness of a healthcare system for depressive disorders by implementing telemedicine: a health economic modeling study. The American journal of geriatric psychiatry : official journal of the American Association for Geriatric Psychiatry. 2014;22(3):253-62.

116. Meijboom MJ, Rozenbaum MH, Benedictus A, Luytjes W, Kneyber MC, Wilschut JC, et al. Cost-effectiveness of potential infant vaccination against respiratory syncytial virus infection in The Netherlands. Vaccine. 2012;30(31):4691-700.

117. Mohseninejad L, Feenstra T, van der Horst HE, Woutersen-Koch H, Buskens E. Targeted screening for Coeliac Disease among irritable bowel syndrome patients: analysis of cost-effectiveness and value of information. The European journal of health economics : HEPAC : health economics in prevention and care. 2013;14(6):947-57.

118. Van Kessel K.E.M KLCDB-GEWZTCMVYZECSEW. FGFR3 mutation analysis in voided urine samples to decrease cystoscopies and cost in nonmuscle invasive bladder cancer surveillance: A comparison of 3 strategies. Journal of Urology. 2013;189(5):1676-1681.

119. van Steensel FJA, Dirksen, C.D., Bögels, S.M. Cost-effectiveness of cognitive-behavioral therapy versus treatment as usual for anxiety disorders in children with autism spectrum disorder. Research in Autism Spectrum Disorders. 2014;8(2).

120. de Bock G H VKMJLOJCSSDMDFTHNGMJ. Which screening strategy should be offered to women with BRCA1 or BRCA2 mutations? A simulation of comparative cost-effectiveness. British Journal of Cancer. 2013;108(8):1579-1586.

121. Boberg KM, Wisloff T, Kjollesdal KS, Stovring H, Kristiansen IS. Cost and health consequences of treatment of primary biliary cirrhosis with ursodeoxycholic acid. Alimentary pharmacology & therapeutics. 2013;38(7):794-803.

122. Oppong R, Jit M, Smith RD, Butler CC, Melbye H, Molstad S, et al. Cost-effectiveness of point-of-care C-reactive protein testing to inform antibiotic prescribing decisions. The British journal of general practice : the journal of the Royal College of General Practitioners. 2013;63(612):e465-71.

123. Carrera-Hueso FJ, Ferriols-Lisart, R., Carrera-Hueso, J.A., Poquet Jornet, J.E., Escoms-Moreno, C., Ramon-Barrios, A., Hernandez Perez, M. . Cost-effectiveness of doripenem or imipenem in ventilator-associated pneumonia. PharmacoEconomics: Spanish Research Articles. 2013;10(4):131-40.

124. Blasco AJ, Arribas JR, Boix V, Clotet B, Domingo P, Gonzalez-Garcia J, et al. [Costs and cost-efficacy analysis of the preferred treatments by GESIDA/National Plan for AIDS for the initial antiretroviral therapy in adult human immunodeficiency virus (HIV) infected patients in 2012]. Enfermedades infecciosas y microbiologia clinica. 2012;30(6):283-93.

125. Díaz MQ, Borobia, A. M., Cachafeiro, S. P., Rodríguez, C., Erce, J. A. G. Cost-effectiveness of using prothrombin complex concentrate to prevent complications related to overdoses of anticoagulants in the emergency department. Emergencias 2012;24(2):113-20.

126. Rubio-Valera M, Bosmans J, Fernandez A, Penarrubia-Maria M, March M, Trave P, et al. Cost-effectiveness of a community pharmacist intervention in patients with depression: a randomized controlled trial (PRODEFAR Study). PloS one. 2013;8(8):e70588.

127. Ohsfeldt RL, Olsson AG, Jensen MM, Gandhi SK, Paulsson T. Cost-effectiveness of rosuvastatin 20 mg for the prevention of cardiovascular morbidity and mortality: a Swedish economic evaluation of the JUPITER trial. Journal of medical economics. 2012;15(1):125-33.

128. Wu DB, Rinaldi F, Huang YC, Chang JA, Chang CJ. Economic evaluation of universal 7-valent pneumococcal conjugate vaccination in Taiwan: a cost-effectiveness analysis. Journal of the Formosan Medical Association = Taiwan yi zhi. 2013;112(3):151-60.

129. Ara RLLMMAFRAMKNMKA. What is the clinical effectiveness and cost-effectiveness of using drugs in treating obese patients in primary care? A systematic review. Health Technology Assessment. 2012;16(5):1-202.

130. Brown T, Pilkington G, Bagust A, Boland A, Oyee J, Tudur-Smith C, et al. Clinical effectiveness and cost-effectiveness of first-line chemotherapy for adult patients with locally advanced or metastatic non-small cell lung cancer: a systematic review and economic evaluation. Health Technol Assess. 2013;17(31):1-278.

131. Carroll C, Hummel S, Leaviss J, Ren S, Stevens JW, Everson-Hock E, et al. Clinical effectiveness and cost-effectiveness of minimally invasive techniques to manage varicose veins: a systematic review and economic evaluation. Health Technol Assess. 2013;17(48):i-xvi, 1-141.

132. Ewer AK, Furmston AT, Middleton LJ, Deeks JJ, Daniels JP, Pattison HM, et al. Pulse oximetry as a screening test for congenital heart defects in newborn infants: a test accuracy study with evaluation of acceptability and cost-effectiveness. Health Technology Assessment. 2012;16(2):1-+.

133. Mavranezouli I, Meader N, Cape J, Kendall T. The cost effectiveness of pharmacological treatments for generalized anxiety disorder. PharmacoEconomics. 2013;31(4):317-33.

134. Miners A, Harris J, Felix L, Murray E, Michie S, Edwards P. An economic evaluation of adaptive e-learning devices to promote weight loss via dietary change for people with obesity. BMC health services research. 2012;12:190.

135. O'Cathail SM, Shaboodien R, Mahmoud S, Carty K, O'Sullivan P, Blagden S, et al. Intravenous versus oral dexamethasone premedication in preventing Paclitaxel infusion hypersensitivity reactions in gynecological malignancies. International journal of gynecological cancer : official journal of the International Gynecological Cancer Society. 2013;23(7):1318-25.

136. Svedbom A, Alvares L, Cooper C, Marsh D, Strom O. Balloon kyphoplasty compared to vertebroplasty and nonsurgical management in patients hospitalised with acute osteoporotic vertebral compression fracture: a UK cost-effectiveness analysis. Osteoporosis international : a journal established as result of cooperation between the European Foundation for Osteoporosis and the National Osteoporosis Foundation of the USA. 2013;24(1):355-67.

137. Allen J.K DHCRSSLFKD. Cost-effectiveness of nurse practitioner/community health worker care to reduce cardiovascular health disparities. Journal of Cardiovascular Nursing. 2013;None(None):None.

138. Clark F, Jackson J, Carlson M, Chou CP, Cherry BJ, Jordan-Marsh M, et al. Effectiveness of a lifestyle intervention in promoting the well-being of independently living older people: results of the Well Elderly 2 Randomised Controlled Trial. Journal of epidemiology and community health. 2012;66(9):782-90.

139. Folse HJ, Green LE, Kress A, Allman R, Dinh TA. Cost-effectiveness of a genetic test for breast cancer risk. Cancer prevention research. 2013;6(12):1328-36.

140. Goulart BH, Bensink ME, Mummy DG, Ramsey SD. Lung cancer screening with low-dose computed tomography: costs, national expenditures, and cost-effectiveness. Journal of the National Comprehensive Cancer Network : JNCCN. 2012;10(2):267-75.

141. Jutkowitz E, Gitlin LN, Pizzi LT, Lee E, Dennis MP. Cost effectiveness of a home-based intervention that helps functionally vulnerable older adults age in place at home. Journal of aging research. 2012;2012:680265.

142. Kacker S NPMSWJFKDSRSKKETAA. Economic evaluation of a hypothetical screening assay for alloimmunization risk among transfused patients with sickle cell disease. Transfusion. 2014;None(None):None.

143. Kamel H, Easton JD, Johnston SC, Kim AS. Cost-effectiveness of apixaban vs warfarin for secondary stroke prevention in atrial fibrillation. Neurology. 2012;79(14):1428-34.

144. Mather RC, 3rd, Hug KT, Orlando LA, Watters TS, Koenig L, Nunley RM, et al. Economic evaluation of access to musculoskeletal care: the case of waiting for total knee arthroplasty. BMC musculoskeletal disorders. 2014;15:22.

145. Najafzadeh M MCALLDWSM. Cost-effectiveness of using a molecular diagnostic test to improve preoperative diagnosis of thyroid cancer. Value in Health. 2012;15(8):1005-1012.

146. Reese ES, Daniel Mullins C, Beitelshees AL, Onukwugha E. Cost-effectiveness of cytochrome P450 2C19 genotype screening for selection of antiplatelet therapy with clopidogrel or prasugrel. Pharmacotherapy. 2012;32(4):323-32.

147. Schackman BR, Leff JA, Polsky D, Moore BA, Fiellin DA. Cost-effectiveness of long-term outpatient buprenorphine-naloxone treatment for opioid dependence in primary care. Journal of general internal medicine. 2012;27(6):669-76.

148. Stein JD, Kim DD, Peck WW, Giannetti SM, Hutton DW. Cost-effectiveness of medications compared with laser trabeculoplasty in patients with newly diagnosed open-angle glaucoma. Archives of ophthalmology. 2012;130(4):497-505.

149. Teresi JA, Ramirez M, Remler D, Ellis J, Boratgis G, Silver S, et al. Comparative effectiveness of implementing evidence-based education and best practices in nursing homes: effects on falls, quality-of-life and societal costs. International journal of nursing studies. 2013;50(4):448-63.

150. Wang BC, Wong ES, Alfonso-Cristancho R, He H, Flum DR, Arterburn DE, et al. Cost-effectiveness of bariatric surgical procedures for the treatment of severe obesity. The European journal of health economics : HEPAC : health economics in prevention and care. 2014;15(3):253-63.
